# Persistent Prostate-Specific Antigen Following Radical Prostatectomy for Prostate Cancer and Mortality Risk

**DOI:** 10.1001/jamaoncol.2025.0110

**Published:** 2025-03-13

**Authors:** Derya Tilki, Ming-Hui Chen, Jing Wu, Hartwig Huland, Markus Graefen, Bruce J. Trock, Misop Han, Anthony V. D’Amico

**Affiliations:** 1 Martini-Klinik Prostate Cancer Center, University Hospital Hamburg Eppendorf, Hamburg, Germany; 2 Department of Urology, University Hospital Hamburg-Eppendorf, Hamburg, Germany; 3 Department of Urology, Koc University Hospital, Istanbul, Turkey; 4 Department of Statistics, University of Connecticut, Storrs; 5 Department of Computer Science and Statistics, University of Rhode Island, Kingston; 6 Department of Urology, Brady Urological Institute, Johns Hopkins Medical Institutions, Baltimore, Maryland; 7 Department of Radiation Oncology, Brigham and Women’s Hospital and Dana Farber Cancer Institute, Boston, Massachusetts

## Abstract

**Importance:**

Whether the conventional 1.5-month to 2.0-month time interval following radical prostatectomy (RP) for prostate cancer (PC) is sufficient to accurately document a persistent prostate-specific antigen (PSA) remains unanswered.

**Objective:**

To evaluate the time necessary to accurately document a persistent PSA level after RP.

**Design, Setting, and Participants:**

This cohort study evaluated whether a significant interaction existed between (1) a pre-RP PSA level greater than 20 ng/mL vs 20 ng/mL or less and (2) persistent PSA vs undetectable PSA after RP on PC-specific mortality (PCSM) risk and all-cause mortality (ACM) risk, adjusting for known PC prognostic factors, age at RP, year of RP, and the time-dependent use of post-RP radiation therapy (RT) and androgen deprivation therapy (ADT). Whether an increasing persistent PSA level was associated with a worse prognosis was also investigated. Patients with T1N0M0 to T3N0M0 PC treated with RP between 1992 and 2020 at 2 academic centers were included. Follow-up data were collected until November 2023. Data were analyzed from July 2024 to January 2025.

**Exposure:**

RP.

**Main Outcomes and Measures:**

Adjusted hazard ratio (aHR) of ACM and PCSM risk.

**Results:**

Of 30 461 patients included in the discovery cohort, the median (IQR) age was 64 (59-68) years; of 12 837 patients included in the validation cohort, the median (IQR) age was 59 (54-64) years. Compared with patients with undetectable PSA, among patients with persistent PSA, a pre-RP PSA level greater than 20 ng/mL vs 20 ng/mL or less was significantly associated with reduced ACM risk (aHR, 0.69; 95% CI, 0.51-0.91; *P* = .01; *P* for interaction < .001) and PCSM risk (aHR, 0.41; 95% CI, 0.25-0.66; *P* < .001; *P* for interaction = .02). This result remained after adjustment for prostate volume and was confirmed in the validation cohort for PCSM risk and may represent a higher proportion of patients with a pre-RP PSA greater than 20 ng/mL vs 20 ng/mL or less who could have reached an undetectable PSA level if additional time for PSA assessment occurred before initiating post-RP therapy for presumed persistent PSA. Notably, there was more frequent and a shorter median time to post-RP RT plus ADT or ADT use in patients with a pre-RP PSA greater than 20 ng/mL (244 of 446 [54.7%] at a median [IQR] of 2.68 [1.51-4.40] months) vs 20 ng/mL or less (338 of 972 [34.8%] at a median [IQR] of 3.30 [2.00-5.39] months). These treatment times were shorter than the times to an undetectable PSA in observed patients (median [IQR] of 2.96 [1.84-3.29] months vs 3.37 [2.35-4.09] months, respectively). Increasing persistent PSA level was associated with an increased ACM risk (aHR, 1.14; 95% CI, 1.04-1.24; *P* = .004) and PCSM risk (aHR, 1.27; 95% CI, 1.12-1.45; *P* < .001).

**Conclusions and Relevance:**

PSA level assessed for at least 3 months after RP may minimize overtreatment, and in this study, a higher persistent PSA level was associated with a worse prognosis.

## Key Points

**Question:**

How long should prostate-specific antigen (PSA) be monitored following radical prostatectomy for prostate cancer (PC) to accurately document a persistent PSA, and does an increasing level portend a worse prognosis?

**Findings:**

In this cohort study of 43 298 patients with PC, among patients with a persistent PSA level but not those with an undetectable PSA level, a pre–radical prostatectomy (RP) PSA level greater than 20 ng/mL vs 20 ng/mL or less was significantly associated with reduced all-cause and PC-specific mortality risk, which can be explained by a higher proportion of patients with a pre-RP PSA level greater than 20 ng/mL vs 20 ng/mL or less who could have reached an undetectable PSA level if additional time for PSA assessment occurred before initiating post-RP therapy for a presumed persistent PSA.

**Meaning:**

PSA level should be assessed for at least 3 months postoperatively to minimize overtreatment, and a higher persistent PSA level was associated with a worse prognosis.

## Teaser

This cohort study evaluates the time necessary to accurately document a persistent prostate-specific antigen level after radical prostatectomy.

## Introduction

A persistent prostate-specific antigen (PSA) following radical prostatectomy (RP) for prostate cancer (PC) is associated with a worse prognosis compared with achieving an undetectable PSA level.^1,2^ While 1.5 to 2.0 months following RP is considered an acceptable interval to assess for persistent PSA,^3^ it may be too short to allow for clearance of the PSA from the serum, particularly at higher pre-RP PSA levels.^4^ If true, then patients with a higher compared with a lower pre-RP level could have a more favorable prognosis than would be expected and may experience overtreatment when offered post-RP therapy for presumed persistent PSA before the time when an undetectable PSA level could be reached. In addition, it is unknown whether an increasing persistent PSA level portends a worse prognosis Therefore, we used 2 academic center databases to test and validate whether a persistent PSA assessed at approximately 2 months post-RP in patients with pre-RP PSA value greater than 20 ng/mL (to convert to ug/L, multiply by 1) compared with 20 ng/mL or less would be significantly associated with a lower PC-specific mortality (PCSM) risk and all-cause mortality (ACM) risk. We also investigated whether an increasing persistent PSA level would be significantly associated with a higher PCSM and ACM risk. For both questions, we adjusted for known PC prognostic factors, age at RP, year of RP, and the time-dependent use of post-RP therapy, including radiation therapy (RT) and/or androgen deprivation therapy (ADT).

## Methods

### Patient Population, Staging, and Treatment

Between January 28, 1992, and June 19, 2020, patients who underwent RP for clinical stage T1N0M0 to T3N0M0 prostate adenocarcinoma at the University Hospital Hamburg-Eppendorf, Hamburg, Germany, were included in the study cohort. Patients with a pre-RP PSA greater than 20 ng/mL or a biopsy Gleason score of 8 to 10 underwent staging with computerized tomography or magnetic resonance imaging of the abdominal pelvic region and bone scan prior to RP. A pathologist with expertise in genitourinary pathology evaluated the prostatectomy specimens and assigned a prostatectomy T category, Gleason score, margin, and pelvic lymph node status. The validation cohort from Johns Hopkins Medical Institutions consisted of patients treated with RP from 1990 to 2017. In accord with federal and institutional guidelines, patients signed an institutional review board (IRB)–approved, protocol-specific informed consent form permitting collection of deidentified data at baseline and follow-up, which were entered by a clinical research coordinator into a secure, password-protected database. This study was IRB approved by the Ethik-Kommission der Aerztekammer Hamburg.

### Follow-Up

Follow-up started on the day of RP and ended on the date of last follow-up or the date of death, whichever occurred first. The database was last updated on November 30, 2023. During follow-up, patients typically had PSA assessed every 2 to 3 months for the first year and then every 6 months for an additional 4 years and annually thereafter.

### Statistical Analysis

#### Characterization of Persistent PSA, Time to Undetectable PSA, and Distribution and Timing of Post-RP Therapies by Pre-RP PSA Level

Descriptive statistics were used to characterize the distribution, median value and median time following RP to measurement of a persistent PSA level based on the first PSA assessment after RP stratified by a pre-RP level greater than 20 ng/mL vs 20 ng/mL or less. The proportion achieving an undetectable PSA and the median time to an undetectable PSA level among observed patients as well as the median time to and proportion receiving post-RP therapy among treated patients were enumerated and dichotomized across the same pre-RP PSA strata.

#### Comparison of the Distribution of Clinical Factors

Descriptive statistics were used to characterize the distribution of the clinical factors among patients who achieved an undetectable PSA or a persistent PSA at first post-RP assessment dichotomized by the median persistent PSA level. For the continuous factors of age at RP and year of RP, medians and IQRs were provided, whereas for categorical factors, counts and frequencies were given for the following groups: pre-RP PSA (less than 4 ng/mL, 4 to 10 ng/mL, 10 to 20 ng/mL, and greater than 20 ng/mL), prostatectomy Gleason score (6, 7, or 8 to 10) and prostatectomy T category (T2, T3a, and T3b/4), margin status (negative or positive), pelvic lymph node status (negative or positive), and post-RP RT or ADT use. The distribution of continuous factors was compared using a Wilcoxon 2-sample test,^5^ whereas categorical factors comparisons were made using a Mantel-Haenszel χ^2^ metric.^6^

#### PCSM and ACM Adjusted Hazard Ratios

Multivariable Fine and Gray^7^ and Cox^8^ regression were used to evaluate the coprimary end points of PCSM and ACM risk, respectively. Cause of death was assigned by the treating physician. Time zero was the date of RP. Two interaction terms were included in the model: (1) persistent PSA vs undetectable PSA after RP and the pre-RP PSA level greater than 20 ng/mL vs 20 ng/mL or less and (2) persistent PSA vs undetectable PSA post-RP and time-dependent^9^ post-RP RT or ADT use. The inclusion of the first interaction term permitted testing of the hypothesis that among patients with persistent PSA, a pre-RP PSA value greater than 20 ng/mL was significantly associated with a lower PCSM risk and ACM risk compared with 20 ng/mL or less. The second interaction term enabled an evaluation of whether time-dependent RT or ADT use post-RP was significantly associated with higher or lower PCSM risk and ACM risk in patients with an undetectable PSA or persistent PSA post-RP. The models were adjusted for age at RP (continuous) and year of RP (continuous taking on integer values by year), prostatectomy Gleason score (6 [baseline], 7, or 8 to 10), prostatectomy T category (T2/3a [baseline] or T3b/4), margin status (negative [baseline] or positive) and pelvic lymph node status (negative [baseline] or positive), and the time-dependent^9^ post-RP RT or ADT use. For the subset of 29 304 patients (96.2%) who had prostate volume information based on pre-RP transrectal ultrasonography, we repeated the models including prostate volume as a continuous covariate. Cut points for covariates were selected based on consensus guideline risk stratifications.^10^ We did not consider persistent PSA as a time-dependent covariate even though it was ascertained approximately 2 months after time 0 (date of RP) because there were no PCSM or ACM events observed until after the assessment for a persistent PSA.

To test the hypothesis that an increasing level of persistent PSA was significantly associated with an increased PCSM risk and ACM risk, we evaluated persistent PSA level as a continuous covariate and pre-RP PSA level included as a categorical covariate (less than 4 ng/mL, 4 to 10 ng/mL [baseline], 10 to 20 ng/mL, and greater than 20 ng/mL) in the Fine and Gray^7^ and Cox^8^ regression multivariable models, respectively, adjusting for the covariates previously mentioned. We natural log–transformed persistent PSA level to provide a normal distribution of values. An adjusted hazard ratio (aHR) with 95% CIs was calculated for each covariate.

#### Adjusted Estimates of PCSM and ACM

**Figure 1.**
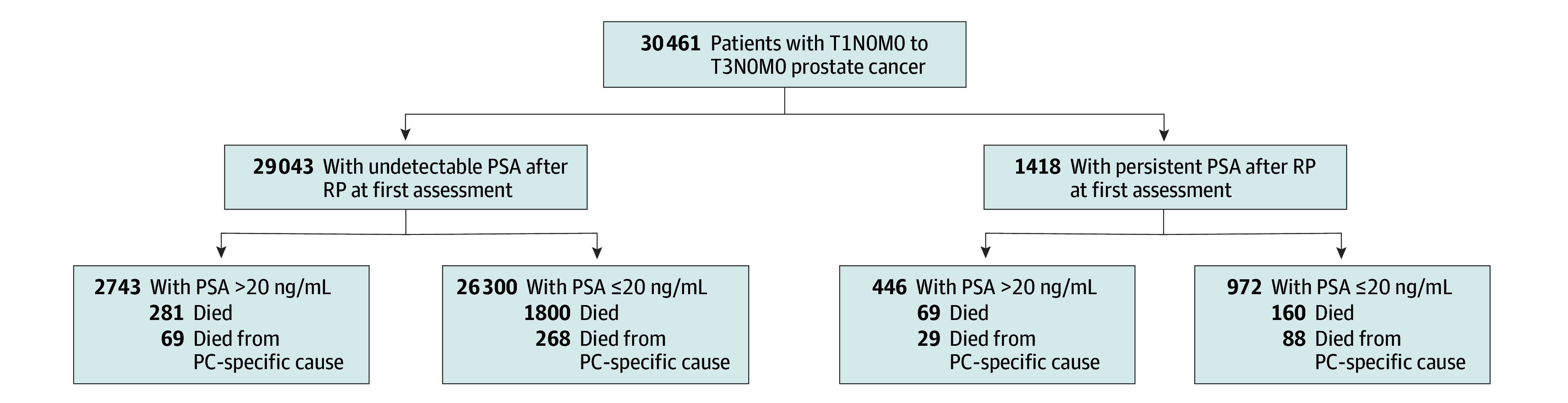
CONSORT Diagram To convert prostate-specific antigen (PSA) to μg/L, multiply by 1. PC indicates prostate cancer; RP, radical prostatectomy.

We used Kaplan-Meier^11^ estimates by reversing the censored indicator as the event indicator to calculate the median follow-up. To illustrate the results of the multivariable model, cumulative incidence^12^ estimates of PCSM and Kaplan-Meier^11^ estimates of overall survival or ACM following RP adjusted for fixed covariates,^13^ and time-dependent covariates^9^ were calculated using the extended Kaplan-Meier methodology with time-dependent covariates^14^ and further adjusted by fixed covariates.^13^ These estimates were then compared using a stratification by the presence or absence of a persistent PSA vs undetectable PSA post-RP in addition to the pre-RP PSA level greater than 20 ng/mL or 20 ng/mL or less, resulting in 4 groups, as shown in the CONSORT diagram in Figure 1. We applied a Bonferroni correction^15^ to adjust for the 4 comparisons such that a 2-sided *P* value less than .0125 (.05/4) was considered statistically significant.

To illustrate the impact that an increasing value of a persistent PSA level after RP had on PCSM risk and ACM risk, we compared the adjusted estimates of PCSM risk and ACM risk in patients with a persistent PSA of 1.0 ng/mL or greater vs less than 1.0 ng/mL, where 1.0 ng/mL corresponded to the 70.5 percentile. SAS version 9.4 (SAS Institute) was used for all calculations except for the covariate adjusted point estimates and 95% CI, where R version 4.4.1 (The R Foundation) was used.

## Results

### Characterization of Persistent PSA, Time to an Undetectable PSA, and Distribution and Timing of Post-RP Therapies by Pre-RP PSA Level

Of 30 461 patients included in the discovery cohort, the median (IQR) age was 64 (59-68) years; of 12 837 patients included in the validation cohort, the median (IQR) age was 59 (54-64) years. As shown in Table 1, of the 30 461 patients in the discovery cohort, 1418 (4.7%) were observed to have a persistent PSA (0.10 ng/mL or greater) at a median (IQR) time from RP to first PSA assessment of 2.17 (1.45-3.02) months. In the validation cohort, 320 (2.5%) had persistent PSA, and 850 (6.6%) died at a median (IQR) follow-up of 5.00 (2.00-12.00) years. As shown in Table 1, there was a significantly higher proportion of patients with a pre-RP PSA greater than 20 ng/mL vs 20 ng/mL or less with a persistent PSA at initial assessment (446 of 3189 [14.0%] vs 972 of 27 272 [3.6%]; *P* < .001) and a higher median (IQR) persistent PSA level (0.65 [0.22-2.04] ng/mL vs 0.30 [0.12-1.00] ng/mL; *P* < .001). Among patients with a PSA level greater than 20 ng/mL vs 20 ng/mL or less, the post-RP PSA values were assessed sooner, with a first assessment following RP at a median (IQR) of 2.00 (1.41-2.99) months and 2.23 (1.45-3.06) months, respectively, and a second assessment at 2.25 (0.79-3.99) months and 2.30 (0.92-4.01) months later. There was more frequent and a shorter median time to post-RP RT plus ADT or ADT use in patients with a pre-RP PSA greater than 20 ng/mL (244 of 446 [54.7%] at a median [IQR] of 2.68 [1.51-4.40] months) vs 20 ng/mL or less (338 of 972 [34.8%] at a median [IQR] of 3.30 [2.00-5.39] months). These median treatment times were shorter than the median (IQR) times to an undetectable PSA level in patients observed for 6 months after RP (median [IQR] of 2.96 [1.84-3.29] months vs 3.37 [2.35-4.09] months, respectively). Also, there was a smaller proportion of patients with a pre-RP PSA level greater than 20 ng/mL compared with 20 ng/mL or less and a persistent PSA post-RP who did not receive post-RP therapy (39 of 446 [8.7%] vs 192 of 972 [19.8%]). However, a higher proportion were observed to experience PCSM, with 8-year adjusted estimates of 13.1% (95% CI, 3.1-47.0) and 5.2% (95% CI, 2.0-13.2), respectively.

**Table 1.** Characterization of Persistent Prostate-Specific Antigen (PSA), Time to an Undetectable PSA, and Distribution and Timing of Post–Radical Prostatectomy (RP) Therapies by Pre-RP PSA Level Among 1418 Patients With a Persistent PSA at the First PSA Assessment Following RP

Characteristic	Pre-RP PSA ≤20 ng/mL	Pre-RP PSA >20 ng/mL	All patients with a persistent PSA
Pre-RP PSA level, median (IQR), ng/mL	10.26 (6.98-13.95)	35.00 (25.60-56.00)	13.51 (8.60-24.77)
Patients with persistent PSA, No./total No. (%)^a^	972/27 272 (3.6)	446/3189 (14.0)	1418/30 461 (4.7)
Persistent PSA level, median (IQR), ng/mL^a^	0.30 (0.12-1.00)	0.65 (0.22-2.04)	0.37 (0.15-1.37)
Time from RP to first assessment for a persistent PSA level, median (IQR), mo^b^	2.23 (1.45-3.06)	2.00 (1.41-2.99)	2.17 (1.45-3.02)
Time between the first and second PSA assessments, median (IQR), mo^b^	2.30 (0.92-4.01)	2.25 (0.79-3.99)	2.30 (0.85-4.01)
Time from RP to an undetectable PSA in patients with a persistent PSA observed for 6 mo following RP^b^			
Median (IQR), mo	3.37 (2.35-4.09)	2.96 (1.84-3.29)	3.22 (2.27-4.04)
No./total No. (%)	68/972 (7.0)	15/446 (3.4)	83/1418 (5.9)
Time to initiation of RT within 1 y of RP for a persistent PSA^b^^,^^c^			
Median (IQR), mo	4.47 (3.42-5.95)	3.84 (3.19-5.03)	4.19 (3.29-5.63)
No./total No. (%)	481/972 (49.5)	279/446 (62.6)	760/1418 (52.9)
Time to initiation of ADT within 1 y of RP for a persistent PSA^b^^,^^d^			
Median (IQR), mo	3.30 (2.00-5.39)	2.68 (1.51-4.40)	3.02 (1.77-5.03)
No./total No. (%)	338/972 (34.8)	244/446 (54.7)	582/1418 (41.0)
Patients receiving ADT plus RT, No./total No. (%)^b^	224/972 (23.1)	165/446 (37.0)	389/1418 (27.4)
Patients with persistent PSA who received RT and/or ADT >1 y following RP, No./total No. (%)^b^	258/972 (26.5)	89/446 (20.0)	347/1418 (24.5)
Patients with a persistent PSA level and no post-RP therapy, No./total No. (%)^b^	192/972 (19.8)	39/446 (8.7)	231/1418 (16.3)

Abbreviations: ADT, androgen deprivation therapy; RT, radiation therapy.

SI conversion factor: To convert PSA to μg/L, multiply by 1.

^a^ *P* < .001.

^b^ Calculation of time-dependent post-RP treatment covariates were made retrospectively given that time 0 was defined as the date of RP.

^c^ Patients could have received RT alone or ADT plus RT.

^d^ Patients could have received ADT alone or ADT plus RT.

### Comparison of the Distribution of Clinical Factors and PC Deaths Among Patients With an Undetectable or Persistent PSA

As shown in Table 2, based on the first PSA assessment after RP, patients with a persistent PSA level up to the median (IQR) value of 0.37 (0.15-1.37) ng/mL were significantly more likely compared with patients with an undetectable PSA level after RP to have a pre-RP PSA level greater than 20 ng/mL, prostatectomy Gleason score 8 to 10, prostatectomy T3b/4, positive surgical margins, and positive pelvic lymph nodes. The same significant results were observed in patients with a persistent PSA value of 0.37 ng/mL or greater vs less than 0.37 ng/mL. These significant trends are reflected in the observation that the proportion of ACM risk that PCSM risk comprised increased for patients with an undetectable PSA vs persistent PSA post-RP from 337 of 2081 (16.2%) to 117 of 229 (51.1%), as shown in Table 2. Moreover, as the persistent PSA level post-RP increased from less than median to above the median value, the proportion of ACM risk due to PCSM risk increased from 31 of 84 (36.9%) to 86 of 145 (59.3%).

**Table 2.** Comparison of Distribution of Clinical Factors Among the 30 461 Study Patients Observed to Have an Undetectable or Persistent Prostate-Specific Antigen (PSA) Level at First PSA Assessment Following Radical Prostatectomy (RP)

Clinical factor	Post-RP PSA, No. (%)			*P* value	
	Undetectable (n = 29 043)	Persistent and below median (n = 706)^a^	Persistent and at or above median (n = 712)^a^	Undetectable vs persistent PSA below median^a^	Persistent PSA below median vs at or above median^a^
Age at RP, median (IQR), y	64 (59-68)	65 (60-69)	64 (59-69)	.001	.38
Year of RP, median (IQR)	2013 (2008-2016)	2014 (2011-2016)	2014 (2011-2016)	<.001	.55
Pre-RP PSA level, median (IQR), ng/mL	7.97 (5.71-12.00)	11.73 (7.28-19.69)	16.50 (10.00-28.93)	<.001	<.001
Prostate volume, median (IQR), mL^b^	39.00 (30.00-52.00)	39.00 (30.00-50.00)	40.85 (32.00-52.00)	.99	.02
Pre-RP PSA level, ng/mL					
<4	2119 (7.3)	37 (5.2)	18 (2.5)	<.001	<.001
4-10	16 945 (58.4%)	257 (36.4)	161 (22.6)		
>10-20	7236 (24.9)	246 (34.8)	253 (35.5)		
>20	2743 (9.4)	166 (23.5)	280 (39.3)		
Prostatectomy Gleason score					
6	4621 (15.9)	44 (6.2)	18 (2.5)	<.001	<.001
7	22 591 (77.8)	505 (71.5)	440 (61.8)		
8-10	1831 (6.3)	157 (22.2)	254 (35.7)		
Prostatectomy T category					
T2	19 060 (65.6)	215 (30.45)	89 (12.5)	<.001	<.001
T3a	6379 (22.0)	189 (26.8)	175 (24.6)		
pT3b/4	3604 (12.4)	302 (42.8)	448 (62.9)		
Margin status					
Negative	23 701 (81.6)	391 (55.4)	306 (43.0)	<.001	<.001
Positive	5342 (18.4)	315 (44.6)	406 (57.0)		
Prostatectomy pelvic lymph node disease					
Negative	26 641 (91.7)	484 (68.6)	333 (46.8)	<.001	<.001
Positive	2402 (8.3)	222 (31.4)	379 (53.2)		
Adjuvant or salvage therapy^c^					
RT	5915 (20.4)	440 (62.3)	440 (61.8)	NA	NA
ADT	3469 (11.9)	321 (45.5)	532 (74.7)	NA	NA
All-cause deaths^c^	2081 (7.2)	84 (11.9)	145 (20.4)	NA	NA
All-cause deaths from prostate cancer^c^	337 (16.2)	31(36.9)	86 (59.3)	NA	NA

Abbreviations: ADT, androgen deprivation therapy; NA, not applicable; RT, radiation therapy.

SI conversion factor: To convert PSA to μg/L, multiply by 1.

^a^ Median value of 1418 patients with a persistent PSA at first assessment was 0.37 ng/mL.

^b^ For the 29 304 patients who had prostate volume information.

^c^ Calculation of the proportions of time-dependent postoperative treatment and deaths were made retrospectively given that time 0 was defined as the date of RP; therefore, *P* values were listed as NA.

### PCSM and ACM aHRs

After a median (IQR) follow-up of 6.23 (3.59-10.25) years, of 30 461 patients, 2310 patients (7.6%) died, and 454 (19.7%) were from PC. As shown in Table 3, a significant interaction was observed between a persistent PSA vs undetectable PSA post-RP and a pre-RP PSA level greater than 20 ng/mL vs 20 ng/mL or less. This significant interaction was reflected in the significant association between a pre-RP PSA level greater than 20 ng/mL vs 20 ng/mL or less in patients with a persistent PSA and a reduced ACM risk (aHR, 0.69; 95% CI, 0.51-0.91; *P* = .01) and PCSM risk (aHR, 0.41; 95% CI, 0.25-0.66; *P* < .001), whereas this was not the case for patients with an undetectable PSA (ACM risk: aHR, 1.18; 95% CI, 1.03-1.34; *P* = .02; PCSM risk: aHR, 0.79; 95% CI, 0.60-1.05; *P* = .10). Increasing prostate volume was not associated with a significant decrease in ACM risk (aHR, 1.00; 95% CI, 0.999-1.001; *P* = .73) or PCSM risk (aHR, 0.99; 95% CI, 0.99-1.00; *P* = .07), and with this covariate included in the models, the significant association between a pre-RP PSA level greater than 20 ng/mL vs 20 ng/mL or less in patients with a persistent PSA and significantly reduced ACM risk (aHR, 0.67; 95% CI, 0.50-0.90; *P* = .01) and PCSM risk (aHR, 0.43; 95% CI, 0.26-0.69; *P* < .001) remained, as it did in the validation cohort for PCSM risk (aHR, 0.26; 95% CI, 0.07-0.88; *P* = .03) but not significantly for ACM risk (aHR, 0.50; 95% CI, 0.18-1.41; *P* = .19). For the PCSM end point, there was significant interaction between the time-dependent use of RT in patients with a persistent PSA vs undetectable PSA post-RP in that RT use was only associated with a significant reduction in PCSM risk among patients with persistent PSA (aHR, 0.54; 95% CI, 0.33-0.87; *P* = .01) but not an undetectable PSA post-RP (aHR, 1.03; 95% CI, 0.78-1.37; *P* = .82).

**Table 3.** Multivariable Interaction Regression Model Adjusted Hazard Ratios (aHRs) for All-Cause Mortality (ACM) and Prostate Cancer–Specific Mortality (PCSM) for Each Clinical Factor Where a Persistent Prostate-Specific Antigen (PSA) at the First PSA Assessment Following Radical Prostatectomy (RP) Was Characterized as a Categorical or Continuous Covariate

Clinical factor	Patients, No.	ACM			PCSM		
		Deaths, No.	aHR (95% CI)^a^	*P* value	PC deaths, No.	aHR (95% CI)^a^	*P* value
**Persistent PSA as a categorical covariate**							
Interaction term: persistent vs undetectable post-RP PSA × pre-RP PSA >20 ng/mL vs ≤20 ng/mL	30 461	2310	0.58 (0.43-0.80)	<.001	454	0.52 (0.30-0.90)	.02
Pre-RP PSA >20 ng/mL							
Persistent PSA	446	69	0.98 (0.66-1.44)	.90	29	3.47 (1.54-7.85)	NA
Undetectable PSA	2743	281	1.0 [Reference]	NA	69	1.0 [Reference]	.003
Pre-RP PSA ≤20 ng/mL							
Persistent PSA	972	160	1.68 (1.25-2.25)	<.001	88	6.67 (3.67-12.15)	<.001
Undetectable PSA	26 300	1800	1.0 [Reference]	NA	268	1.0 [Reference]	NA
Post-RP persistent PSA							
Pre-RP PSA >20 ng/mL	446	69	0.69 (0.51-0.91)^b^	.01	29	0.41 (0.25-0.66)^b^	<.001
Pre-RP PSA ≤20 ng/mL	972	160	1.0 [Reference]	NA	88	1.0 [Reference]	NA
Post-RP undetectable PSA							
Pre-RP PSA >20 ng/mL	2743	281	1.18 (1.03-1.34)	.017	69	0.79 (0.60-1.05)	.10
Pre-RP PSA ≤20 ng/mL	26 300	1800	1.0 [Reference]	NA	268	1.0 [Reference]	NA
Adjuvant or salvage therapy use							
Interaction term: persistent vs undetectable post-RP PSA × RT (time-dependent)	6795	613	0.87 (0.64-1.17)	.34	248	0.54 (0.33-0.87)	.01
RT(time-dependent)							
Undetectable post-RP PSA	5915	483	0.76 (0.67-0.87)	<.001	186	1.03 (0.78-1.37)	.82
Persistent post-RP PSA	880	130	0.66 (0.50-0.87)	.003	62	0.55 (0.36-0.86)	.008
Interaction term: persistent vs undetectable post-RP PSA, ng/ml x ADT (time-dependent)	4322	676	1.09 (0.78-1.53)	.62	360	0.41 (0.21-0.82)	.01
ADT (time-dependent)							
Undetectable post-RP PSA	3469	502	1.99 (1.74-2.27)	<.001	253	12.01 (8.33-17.32)	<.001
Persistent post-RP PSA	853	174	2.17 (1.57-2.99)	<.001	104	4.96 (2.69-9.15)	<.001
**Persistent PSA as a continuous covariate**							
Log-transformed persistent post-RP PSA	1418	229	1.14 (1.04-1.24)	.004	117	1.27 (1.12-1.45)	<.001
Adjuvant or salvage therapy							
RT (time-dependent)	880	130	0.55 (0.41-0.76)	<.001	62	0.52 (0.33-0.83)	.006
ADT (time-dependent)	853	174	1.74 (1.22-2.49)	.002	104	4.33 (2.25-8.33)	<.001

Abbreviations: ADT, androgen deprivation therapy; NA, not applicable; RT, radiation therapy.

SI conversion factor: To convert PSA to μg/L, multiply by 1.

^a^ The models were adjusted for age at RP, year of RP, prostatectomy T category, prostatectomy Gleason score, margin, and pelvic lymph node status. See the eTable in Supplement 1 for results of fixed covariates.

^b^ Including prostate volume in the models did not change the direction or significance of the effects (ie, aHR <1.00).

After a median (IQR) follow-up of 6.05 (3.56-9.07) years, of 1418 patients with persistent PSA after RP, 229 (16.2%) died, and 117 deaths (51.1%) were from PC. As shown in Table 3, an increasing persistent PSA level was significantly associated with increased ACM risk (aHR, 1.14; 95% CI, 1.04-1.24; *P* = .004) and PCSM risk (aHR, 1.27; 95% CI, 1.12-1.45; *P* < .001), whereas for patients with a pre-RP PSA greater than 20 ng/mL, these risks were reduced (ACM risk: aHR, 0.71; 95% CI, 0.49-1.04; *P* = .08; PCSM risk: aHR, 0.44; 95% CI, 0.24-0.71; *P* = .002). See the eTable in Supplement 1 for the complete results of the fixed covariates for the models where the persistent PSA level was characterized as a categorical as well as continuous covariate.

### Adjusted Estimates of PCSM and ACM

**Figure 2.**
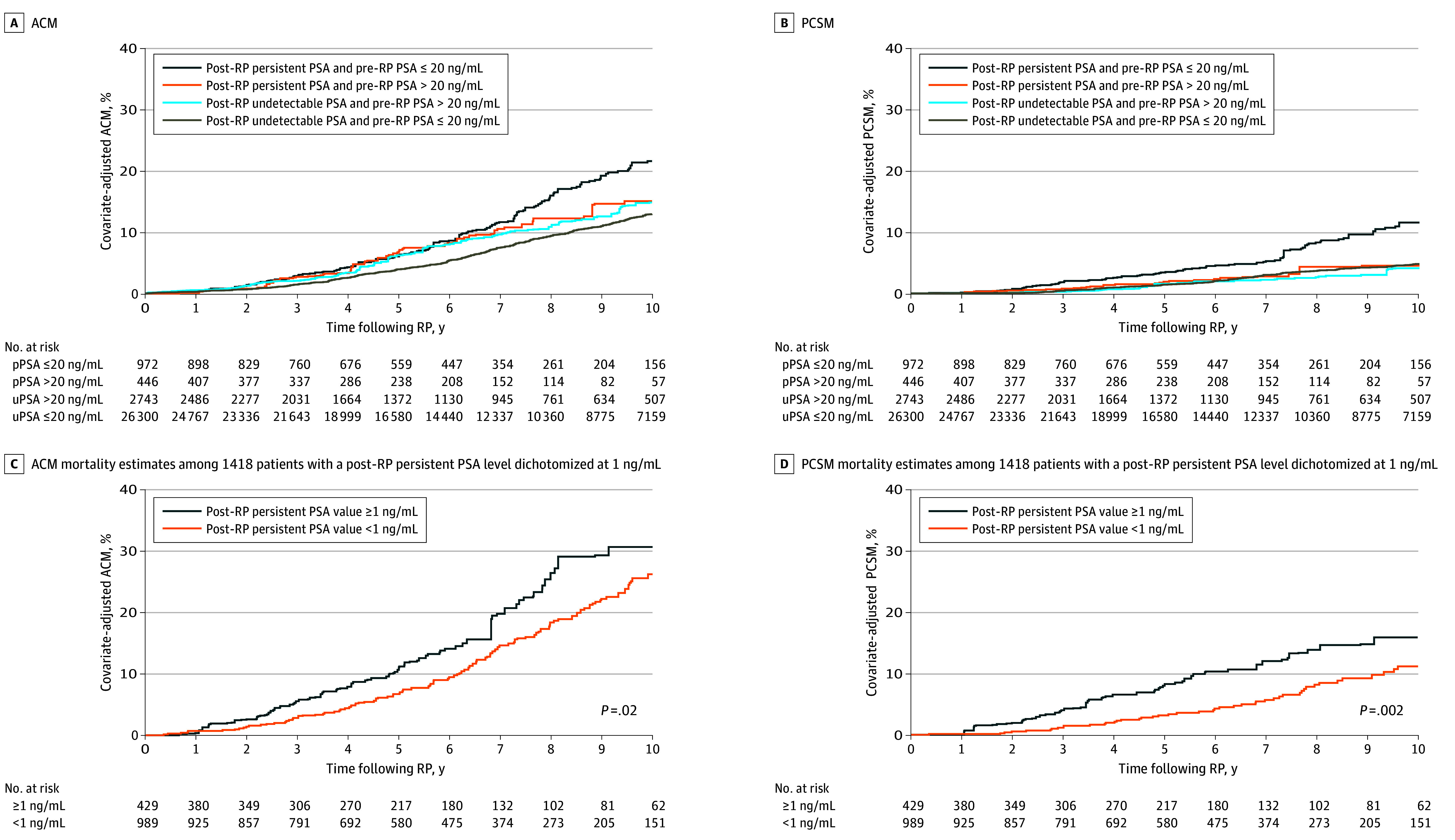
Adjusted All-Cause Mortality (ACM) and Prostate Cancer–Specific Mortality (PCSM) Estimates A and B, Adjusted Kaplan-Meier estimates of ACM and cumulative incidence estimates of PCSM for 30 461 patients. C and D, Adjusted Kaplan-Meier estimates of ACM and cumulative incidence estimates of PCSM for 1418 patients with a post–radical prostatectomy (RP) persistent prostate-specific antigen (PSA), which was defined at time of first PSA assessment following RP. Estimates were adjusted for known prostate cancer prognostic factors, age at RP, year of RP, and the time-dependent use of post-RP radiation therapy and/or androgen deprivation therapy. To convert PSA to μg/L, multiply by 1. pPSA indicates persistent prostate-specific antigen; uPSA, undetectable prostate-specific antigen.

As shown in Figure 2A and B, after adjustment for multiple comparisons, patients with a persistent PSA post-RP and a pre-RP PSA level greater than 20 ng/mL vs 20 ng/mL or less had significantly lower estimates of ACM risk and PCSM risk, whereas this was not true for patients with an undetectable PSA after RP. Specifically, for patients with a persistent PSA level post-RP and a pre-RP PSA greater than 20 ng/mL vs 20 ng/mL or less, 8-year point estimates of ACM were 12.15% (95% CI, 9.25-15.87) vs 15.84% (95% CI, 12.99%-19.24%), respectively, whereas these estimates were 10.78% (95% CI, 8.80-13.17) and 9.32% (95% CI, 8.40-10.33), respectively, for patients with an undetectable PSA. With respect to the PCSM risk end point, these 8-year point estimates were 4.33% (95% CI, 2.67-6.99) vs 8.32% (95% CI, 6.70-10.30), respectively, for patients with a persistent PSA post-RP and 2.63% (95% CI, 1.79-3.87) and 3.71% (95% CI, 3.10-4.44), respectively, for patients with an undetectable PSA after RP.

As shown in Figure 2C and D, patients with a persistent PSA level post-RP of 1 ng/mL or greater compared with less than 1 ng/mL had significantly higher estimates of ACM risk and PCSM risk. Specifically, for patients with a post-RP persistent PSA of 1 ng/mL or greater compared with less than 1 ng/mL, 8-year point estimates of ACM risk were 26.49% (95% CI, 21.92-31.80) vs 18.41% (95% CI, 15.24-22.14), respectively. For PCSM risk, these 8-year point estimates were 13.86% (95% CI, 10.47-18.22) vs 8.17% (95% CI, 6.08-10.93), respectively.

## Discussion

In this study, a counterintuitive association was observed between a significantly lower ACM risk and PCSM risk in patients with a persistent PSA assessed at a median of 2.17 months post-RP and a pre-RP PSA greater than 20 ng/mL vs 20 ng/mL or less, and this significant association remained after adjustment for prostate gland volume and was observed in the validation cohort for PCSM risk. To find a reason for the more favorable prognosis in patients with a higher pre-RP PSA level, we hypothesized that a higher proportion of patients with a pre-RP PSA greater than 20 ng/mL compared with 20 ng/mL or less assessed for a persistent PSA at the conventional 1.5-month to 2.0-month time point post-RP could have reached an undetectable PSA level if further PSA assessment was performed before initiating post-RP therapy for a presumed persistent PSA. The evidence to support this hypothesis is shown in Table 1. First, patients with a pre-PSA greater than 20 ng/mL vs 20 ng/mL or less had earlier PSA assessments following RP, which could have prompted physicians to initiate post-RP therapy sooner, given the presumed persistent PSA and the known pre-RP PSA greater than 20 ng/mL. This was substantiated by the more frequent and shorter median time to post-RP RT plus ADT use or ADT use during the first year following RP in patients with a pre-RP PSA level greater than 20 ng/mL compared with 20 ng/mL or less. Moreover, these median treatment times after RP were shorter than the median times to an undetectable PSA in patients observed for 6 months after RP and shorter by a greater amount in patients with a pre-RP PSA level greater than 20 ng/mL compared with 20 ng/mL or less.

The clinical significance of these findings is that they highlight the need to monitor PSA after RP for longer than the commonly practiced 1.5-month to 2.0-month interval before concluding a persistent PSA exists and initiating post-RP therapy to minimize the risk of overtreatment. These findings are relevant in patients who achieve an undetectable PSA with further follow-up and have no or at most 1 high-risk factor at RP (T3 or T4 disease or Gleason score of 8 to 10) given the randomized data suggesting no benefit in metastasis-free survival for adjuvant compared with early salvage RT.^16^ For patients with 2 or more high-risk factors^17^ or pN1 PC,^18^ adjuvant RT may be indicated, given it is associated with reduced ACM risk.

We also found that an increasing post-RP persistent PSA level was significantly associated with increased ACM risk and PSCM risk. Therefore, patients with higher persistent PSA values should be included in future randomized trials evaluating the impact on PCSM and ACM risk when comparing post-RP standard of care treatment with RT and conventional ADT with treatment escalation approaches^19^ using androgen synthesis or receptor inhibitors. Future study is needed to elucidate the predictive capability of genomic classifiers^20^ and artificial intelligence platforms^21^ in identifying patients with a persistent PSA who would benefit from treatment escalation approaches^19^ vs standard of care.

Some results deserve further discussion. First, the shorter distribution of times to an undetectable PSA in observed patients with PSA greater than 20 ng/mL vs 20 ng/mL or less may be explained by the shorter time intervals from RP to PSA assessments in patients with a pre-RP PSA greater than 20 ng/mL. Second, higher PCSM estimates 8 years after RP were observed in patients who did not receive post-RP therapy and had a persistent PSA post-RP and a pre-RP PSA greater than 20 ng/mL compared with 20 ng/mL or less, which should have led to a worse prognosis in patients with a pre-RP PSA greater than 20 ng/mL but the opposite was observed. This reversal of outcome may be explained by a higher proportion of patients with a pre-RP PSA level greater than 20 ng/mL compared with 20 ng/mL or less who were going to achieve an undetectable PSA if observed for a longer period before initiating post-RP therapy. Third, as noted in Table 3, there was a significant association between an increased PCSM risk and ACM risk in patients with a persistent PSA compared with undetectable PSA after RP when the pre-RP PSA was 20 ng/mL or less but not when the pre-RP PSA was greater than 20 ng/mL, where only PCSM risk was significantly increased and to a lesser extent compared with patients with a pre-RP PSA of 20 ng/mL or less, with aHRs of 3.47 (95% CI, 1.54-7.85) vs 6.67 (95% CI, 3.67-12.15), respectively. The smaller increase in the aHR for PCSM risk is consistent with a higher proportion of patients with a persistent PSA and a pre-RP PSA greater than 20 ng/mL vs 20 ng/mL or less who may have achieved an undetectable PSA given longer follow-up before initiating post-RP therapy for presumed persistent PSA. Given the smaller aHR for PCSM risk, translation into a significant increase in ACM risk may not have been possible given the competing risks for mortality in this patient population. Finally, the time-dependent use of RT following RP in patients with an undetectable PSA level was associated with a significantly reduced ACM risk but not PCSM risk. An explanation for this result could be the selection of healthier patients for adjuvant RT, and given the undetectable PSA level, many of these patients may have been cured following RP and were not at risk for PCSM but lived longer based on better overall health.

### Limitations

This study is limited by the validation cohort showing a significant association between a reduction in PCSM risk but not ACM risk in patients with a persistent PSA and pre-RP PSA greater than 20 ng/mL vs 20 ng/mL or less.. This may be explained by the limited power to assess the ACM end point given the shorter median follow-up and smaller event rate in the validation cohort compared with the discovery cohort. However, given the significant reduction in PCSM risk, a significant reduction in ACM risk would be expected with further follow-up in otherwise healthy patients.

## Conclusions

In conclusion, PSA level assessed for at least 3 months after RP may minimize overtreatment, and in this study, a higher persistent PSA level was associated with a worse prognosis.

## Notes

Supplement 1. **eTable.** Multivariable Interaction Regression Model Adjusted Hazard Ratios for Prostate Cancer-Specific and All-Cause Mortality for Each Clinical Factor Where a pPSA at the First PSA Assessment Following RP Was Characterized as a Categorical (2A) or Continuous (2B) Covariate

Supplement 2. Data Sharing Statement
