# Supplementary material for: Persistent Prostate-Specific Antigen Following Radical Prostatectomy for Prostate Cancer and Mortality Risk
Source: JAMA Oncol. 2025 Mar 13;11(5):502–10. doi: 10.1001/jamaoncol.2025.0110 (PMC11907358; doi:10.1001/jamaoncol.2025.0110)
Supplement: Supplement 1. — eTable. Multivariable Interaction Regression Model Adjusted Hazard Ratios for Prostate Cancer-Specific and All-Cause Mortality for Each Clinical Factor Where a pPSA at the First PSA Assessment Following RP Was Characterized as a Categorical (2A) or Continuous (2B) Covariate [file jamaoncol-e250110-s001.pdf]

Supplemental Online Content

Tilki D, Chen MH, Wu J, et al. Persistent prostate-specific antigen following radical prostatectomy for prostate cancer and mortality risk. *JAMA Oncol*. Published online March 13, 2025. doi:10.1001/jamaoncol.2025.0110

eTable. Multivariable Interaction Regression Model Adjusted Hazard Ratios for Prostate Cancer-Specific and All-Cause Mortality for Each Clinical Factor Where a pPSA at the First PSA Assessment Following RP Was Characterized as a Categorical (2A) or Continuous (2B) Covariate

This supplementary material has been provided by the authors to give readers additional information about their work.

© 2025 Tili D et al. *JAMA Oncology*.

eTable. Multivariable Interaction Regression Model Adjusted Hazard Ratios for Prostate Cancer-Specific and All-Cause Mortality for Each Clinical Factor Where a pPSA at the First PSA Assessment Following RP Was Characterized as a Categorical (2A) or Continuous (2B) Covariate

A

Clinical Factor	Number of patients	ACM			PCSM		
		Number of Deaths	AHR [95% CI]	p-value	Number of PC deaths	AHR [95% CI]	p-value
**Interaction Term:**<br><br>**Persistent vs Undetectable post-RP PSA in ng/ml x pre-RP PSA > vs ≤ 20 ng/ml**	30461	2310	0.58 (0.43, 0.80)	<0.001	454	0.52 (0.30, 0.90)	0.02
**pre-RP PSA > 20 ng/ml**<br><br>**Persistent PSA**	446	69	0.98	0.90	29	3.47	0.003

© 2025 Tili D et al. *JAMA Oncology*.

**Undetectable PSA**	2743	281	(0.66, 1.44)<br>1.0 (Ref)	-	69	(1.54, 7.85)<br>1.0 (Ref)	-
**pre-RP PSA ≤ 20 ng/ml**							
**Persistent PSA**	972	160	1.68 (1.25, 2.25)	<0.001	88	6.67 (3.67, 12.15)	<0.001
**Undetectable PSA**	26300	1800	1.0 (Ref)	-	268	1.0 (Ref)	-
**Post-RP Persistent PSA**							
**pre-RP PSA > 20 ng/ml**	446	69	*0.69 (0.51, 0.91)	0.01	29	*0.41 (0.25, 0.66)	<0.001
**pre-RP PSA ≤ 20 ng/ml**	972	160	1.0 (Ref)	-	88	1.0 (Ref)	-

© 2025 Tili D et al. *JAMA Oncology*.

**Post-RP Undetectable PSA**							
**Pre-RP PSA > 20 ng/ml**	2743	281	1.18 (1.03, 1.34)	0.017	69	0.79 (0.60, 1.05)	0.10
**Pre- RP PSA ≤ 20 ng/ml**	26300	1800	1.0 (Ref)	-	268	1.0 (Ref)	-
**Age (continuous) at RP in Years**	30461	2310	1.07 (1.06, 1.07)	<0.001	454	1.00 (0.98, 1.02)	0.92
**Year (continuous) of RP**	30461	2310	0.964 (0.956, 0.973)	<0.001	454	0.89 (0.88, 0.91)	<0.001
**pGleason score**							
**6 (baseline)**	4683	422	1.0 (Ref)	-	17	1.0 (Ref)	-
**7**	23536	1531	1.27 (1.13, 1.43)	<0.001	258	2.63 (1.56, 4.43)	<0.001

© 2025 Tili D et al. *JAMA Oncology*.

**8-10**	2242	357	2.51 (2.09, 3.01)	<0.001	179	7.56 (4.21, 13.56)	<0.001
**pT category**							
**T2/3a (baseline)**	26107	1665	1.0 (Ref)	-	162	1.0 (Ref)	-
**T3b/4**	4354	645	1.40 (1.24, 1.58)	<0.001	292	2.05 (1.58, 2.64)	<0.001
**Margin status**							
**Negative (baseline)**	24398	1632	1.0 (Ref)	-	225	1.0 (Ref)	-
**Positive**	6063	678	1.28 (1.16, 1.42)	<0.001	229	1.33 (1.07, 1.65)	0.01
**pN1**							
**Negative (baseline)**	27458	1907	1.0 (Ref)	-	273	1.0 (Ref)	-
**Positive**	3003	403	1.37		181	1.40	0.01

© 2025 Tili D et al. *JAMA Oncology*.

			(1.19, 1.57)	<0.001		(1.08, 1.82)	
**Adjuvant or Salvage Therapy**							
**Interaction Term: Persistent vs Undetectable post-RP PSA in ng/ml x RT (t)**	6795	613	0.87 (0.64, 1.17)	0.34	248	0.54 (0.33, 0.87)	0.01
**RT(t)**							
**Undetectable post-RP PSA**	5915	483	0.76 (0.67, 0.87)	<0.001	186	1.03 (0.78, 1.37)	0.82
**Persistent post-RP PSA**	880	130	0.66 (0.50, 0.87)	0.003	62	0.55 (0.36, 0.86)	0.008

© 2025 Tili D et al. *JAMA Oncology*.

**Interaction Term: Persistent vs Undetectable post-RP PSA in ng/ml x ADT (t)**	4322	676	1.09 (0.78, 1.53)	0.62	360	0.41 (0.21, 0.82)	0.01
**ADT (t)**							
**Undetectable post-RP PSA**	3469	502	1.99 (1.74, 2.27)	<0.001	253	12.01 (8.33, 17.32)	<0.001
**Persistent post-RP PSA**	853	174	2.17 (1.57, 2.99)	<0.001	104	4.96 (2.69, 9.15)	<0.001

*Including prostate volume in cc in the models did not change the direction or significance of the effects (i.e. AHR < 1.00)

ACM: All-Cause Mortality; PCSM: Prostate Cancer-Specific Mortality; PC: prostate cancer: AHR: Adjusted Hazard Ratio; CI: Confidence Interval; RP: radical prostatectomy; ng/ml: nanograms/milliliter; Ref: Reference; N1: pelvic lymph node; (t): time-dependent covariate

© 2025 Tili D et al. *JAMA Oncology*.

B

Clinical Factor	Number of patients	ACM			PCSM		
		Number of Deaths	AHR [95% CI]	p-value	Number of PC deaths	AHR [95% CI]	p-value
**Log Persistent post-RP PSA in ng/ml (continuous)**	1418	229	1.14 (1.04, 1.24)	0.004	117	1.27 (1.12, 1.45)	<0.001
**Age (continuous) at RP in Years**	1418	229	1.00 (0.98, 1.03)	0.69	117	1.01 (0.97, 1.04)	0.74
**Year (continuous) of RP**	1418	229	0.98 (0.95, 1.01)	0.13	117	0.92 (0.88, 0.95)	<0.001
**Pre-RP PSA in ng/ml**			1.76			1.92	

© 2025 Tili D et al. *JAMA Oncology*.

**< 4**	55	12	(0.94, 3.31)	0.08	6	(0.81, 4.56)	0.14
**4- 10**	418	58	1.0 (Ref)	-	30	1.0 (Ref)	-
**> 10 -20**	499	90	1.19 (0.85, 1.67)	0.31	52	1.08 (0.67, 1.73)	0.75
**>20**	446	69	0.71 (0.49, 1.04)	0.08	29	0.41 (0.24, 0.71)	0.002
**pGleason score**							
**6 (baseline)**	62	6	1.0 (Ref)	-	1	1.0 (Ref)	-
**7**	945	129	2.55 (1.08, 6.03)	0.03	53	5.41 (0.76, 38.35)	0.09
**8-10**	411	94	4.57 (1.85, 11.28)	0.001	63	15.34 (2.09, 112.38)	0.007
**pT category**							
**T2/3a (baseline)**	668	76	1.0 (Ref)	-	34	1.0 (Ref)	-

© 2025 Tili D et al. *JAMA Oncology*.

**T3b/4**	750	153	1.46 (1.05, 2.01)	0.02	83	1.33 (0.83, 2.13)	0.23
**Margin status** **Negative (baseline)**	697	92	1.0 (Ref)	-	44	1.0 (Ref)	-
**Positive**	721	137	1.63 (1.21, 2.20)	0.001	73	1.64 (1.06, 2.52)	0.03
**pN1** **Negative (baseline)**	817	121	1.0 (Ref)	-	56	1.0 (Ref)	-
**Positive**	601	108	1.19 (0.87, 1.63)	0.27	61	1.32 (0.84, 2.07)	0.24
**Adjuvant or Salvage Therapy**** **RT (t)**	880	130	0.55	<0.001	62	0.52	0.006

© 2025 Tili D et al. *JAMA Oncology*.

			(0.41, 0.76)			(0.33, 0.83)	
**ADT (t)**	853	174	1.74 (1.22, 2.49)	0.002	104	4.33 (2.25, 8.33)	<0.001

ACM: All-Cause Mortality; PCSM: Prostate Cancer-Specific Mortality; PC: prostate cancer: AHR: Adjusted Hazard Ratio; CI: Confidence Interval; RP: radical prostatectomy; ng/ml: nanograms/milliliter; T: tumor category, p: prostatectomy, Ref: Reference: N1: pelvic lymph node; (t): time-dependent covariate

© 2025 Tili D et al. *JAMA Oncology*.
