# Supplementary material for: Persistent Prostate-Specific Antigen Following Radical Prostatectomy for Prostate Cancer and Mortality Risk
Source: JAMA Oncol. 2025 Mar 13;11(5):502–10. doi: 10.1001/jamaoncol.2025.0110 (PMC11907358; doi:10.1001/jamaoncol.2025.0110)
Supplement: Supplement 2. — Data Sharing Statement [file jamaoncol-e250110-s002.pdf]

Data Sharing Statement

Tilki. Persistent Prostate-Specific Antigen Following Radical Prostatectomy for Prostate Cancer and Mortality Risk. *JAMA Oncol*. Published March 13, 2025. doi:10.1001/jamaoncol.2025.0110

Data
Data available: No

Additional Information
Explanation for why data not available: Deidentified data used for the analysis in the current study can be made available upon request from the first author (Dr. Derya Tilki).
